# Towards do-it-yourself planar optical components using plasmon-assisted etching

**DOI:** 10.1038/ncomms10468

**Published:** 2016-01-27

**Authors:** Hao Chen, Abdul M. Bhuiya, Qing Ding, Harley T. Johnson, Kimani C. Toussaint Jr

**Affiliations:** 1 Department of Mechanical Science and Engineering, University of Illinois Urbana-Champaign, Urbana, Illinois 61801, USA; 2 Department of Electrical and Computer Engineering, University of Illinois Urbana-Champaign, Urbana, Illinois 61801, USA

## Abstract

In recent years, the push to foster increased technological innovation and basic scientific and engineering interest from the broadest sectors of society has helped to accelerate the development of do-it-yourself (DIY) components, particularly those related to low-cost microcontroller boards. The attraction with DIY kits is the simplification of the intervening steps going from basic design to fabrication, albeit typically at the expense of quality. We present herein plasmon-assisted etching as an approach to extend the DIY theme to optics, specifically the table-top fabrication of planar optical components. By operating in the design space between metasurfaces and traditional flat optical components, we employ arrays of Au pillar-supported bowtie nanoantennas as a template structure. To demonstrate, we fabricate a Fresnel zone plate, diffraction grating and holographic mode converter—all using the same template. Applications to nanotweezers and fabricating heterogeneous nanoantennas are also shown.

## Abstract (web summary)

Recently, there has been a growing interest in do-it-yourself components to accelerate development of inexpensive fabrication approaches. Here, Chen *et al.* present a plasmon-assisted etching technique to fabricate planar optical components using arrays of gold pillar-supported bowtie nanoantennas as a template.

## Graphical abstract

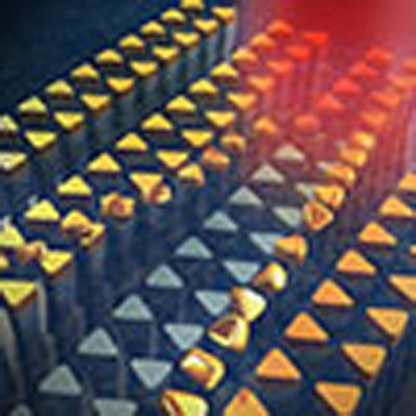

The maker movement has gained momentum in recent years thanks in large part to the reduction in cost of three-dimensional printers and the concomitant rise of inexpensive, do-it-yourself (DIY) microcontroller boards such as those made by Arduino and Raspberry Pi^1^,^2^. A strong theme with this movement is that reducing the number of steps in the manufacturing process, for example, from original equipment manufacturing to actual end product, could help spur learning and innovation, as well as potentially transform existing industries or usher in new ones, thereby leading to economic growth. This concept has even been extended to the production of low-cost DIY atomic force microscopes^3^. Notwithstanding the impressive range of activities and projects that have resulted from DIY kits, this trend has yet to lead to the realization of basic DIY optical components such as lenses or diffraction gratings. A major reason is because inexpensive additive manufacturing approaches result in effectively non-functional optical elements due to the inherent surface roughness between the various added layers. Interestingly, a hint towards realizable DIY optical components possessing basic functionality is to use planar structures based on either diffractive optical elements (DOEs) or metasurfaces^4^,^5^,^6^,^7^,^8^. DOEs are the state-of-the-art and utilize surface features on the order of the wavelength of light, in typically millimetre-thick plastic or quartz, to impart the desired phase onto an optical field. Moreover, advances in fabrication have made the design iteration step increasingly practical. DOEs are primarily limited by their operating bandwidth. Alternatively, metasurfaces^4^,^5^,^6^,^7^,^8^ have shown to alter the properties of light with the added advantage of being even more ultrathin and lightweight^9^. In addition, by using artificial resonators, the constituent subwavelength nanoantennas, relatively broadband operation has been demonstrated^10^,^11^. However, current fabrication approaches are complicated by the fact that the feedback loop from design-to-fabrication-to-application is slow and non-trivial^6^,^7^. This limits quick testing and improvement on an initial design without having to begin anew each time in the cleanroom.

In this paper, we show how arrays of Au pillar-supported bowtie nanoantennas (pBNAs) can be fabricated once in a cleanroom and subsequently used as a template that enables table-top fabrication of multiple, planar optical components using laser-scanning optical microscopy^12^,^13^. This specialized template can be used to short-circuit the design iteration steps, obviating the need for in-depth knowledge of the phase-modifying behaviour of the constituent nanoparticles^12^,^13^,^14^. Thus, we demonstrate the table-top fabrication of a diffraction grating, Fresnel zone plate (FZP), and a holographic mode converter for generating orbital angular momentum—all using the same template. To achieve this streamlining in fabrication, we sacrifice the subwavelength sculpting of the optical wavefront offered by metasurfaces with one that is diffraction-limited, which is sufficient for many basic applications. We show that the enhanced local heating from plasmonics can enable facile table-top plasmon-assisted etching (PAE) of metal. We also demonstrate that PAE can be used to tune the radial extent of near-field trapping forces of nanotweezers^15^,^16^, and offers a promising route to readily engineering novel nanoantenna arrays that are heterogeneous in both space and material composition^17^,^18^,^19^,^20^.

## Results

### Understanding plasmon-assisted etching

**Figure 1:**
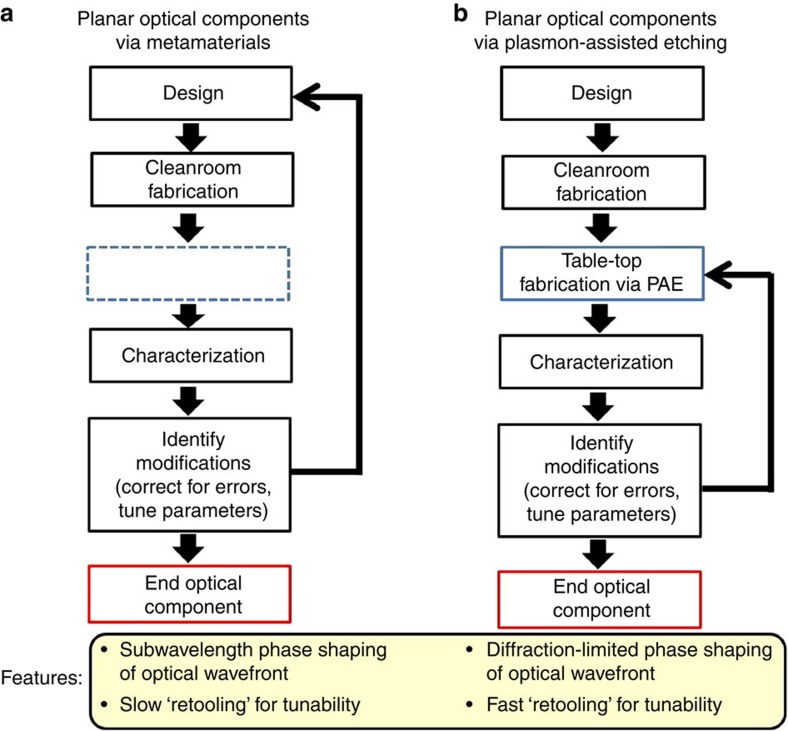
Fabrication process of planar optical components. A flow diagram comparing the fabrication process of planar optical components using a(**a**) metasurface approach and (**b**) a PAE approach.

For DIY optics that may be based on the use of planar optical components, metasurfaces are arguably the emerging technology, whereby almost any desired planar optical component can be fabricated. This is especially attractive for applications where both small form factors and nearly negligible mass are sought while maintaining exquisite control of the optical field. Indeed, previous experiments have demonstrated the capability of metasurfaces in fabricating various planar optical components, such as lenses^21^,^22^,^23^, blazed gratings^8^,^24^,^25^, holographic plates^26^, polarizers and wave plates^27^,^28^. In general, metasurfaces provide subwavelength control of the field, by way of judiciously placed nanoantennas, at the expense of an overall complex fabrication process that is slow to adapt to desired changes in end functionality or corrections to errors—a feature that hampers potential application to DIY optics. For example, designing metasurface-based flat lenses of different focal lengths requires first computing for each lens the required phase relationships for the nanostructures and then fabricating each in a cleanroom. PAE provides a complementary approach to fabricating planar optical components by eliminating the need to go back to the cleanroom and rather instead using a one-time fabricated nanoantenna template^12^. A flow diagram comparing our PAE approach to a metasurface-based method for fabricating planar optical components is depicted in Fig. 1. Here, we observe that metasurface fabrication described in Fig. 1a begins at the design stage, whereby a particular arrangement of nanoantennas must be computed for a target functionality, for example, focusing light to a specific distance. The design is then taken for fabrication in the cleanroom and subsequently subject to various characterization experiments. Based on these experiments the last stage could be the realization of the desired planar optical component. However, if characterization reveals errors in the component, or if a particular parameter needs to be tuned, then the entire process has to begin anew from the basic design stage. Now let us evaluate the PAE process described by Fig. 1b. The first step is to establish a template, which for the work carried out here is based on the use of Au pBNAs. The pBNAs template is then fabricated in the cleanroom. Next, the template is taken to a laser-scanning optical microscope, whereby spatially directed pulsed laser illumination is used to debond the Au nanoantennas from their silica pillars in a desired pattern. The fabricated structure can then be characterized and tested for errors. If there are errors or a need to change the parameters of the fabricated component, then the process goes back to the table-top fabrication stage. This is a significant difference compared with the metasurface approach. As a result, PAE offers a more intuitive, fast, and reconfigurable fabrication process with the tradeoff of diffraction-limited shaping of the optical wavefront.

**Figure 2:**
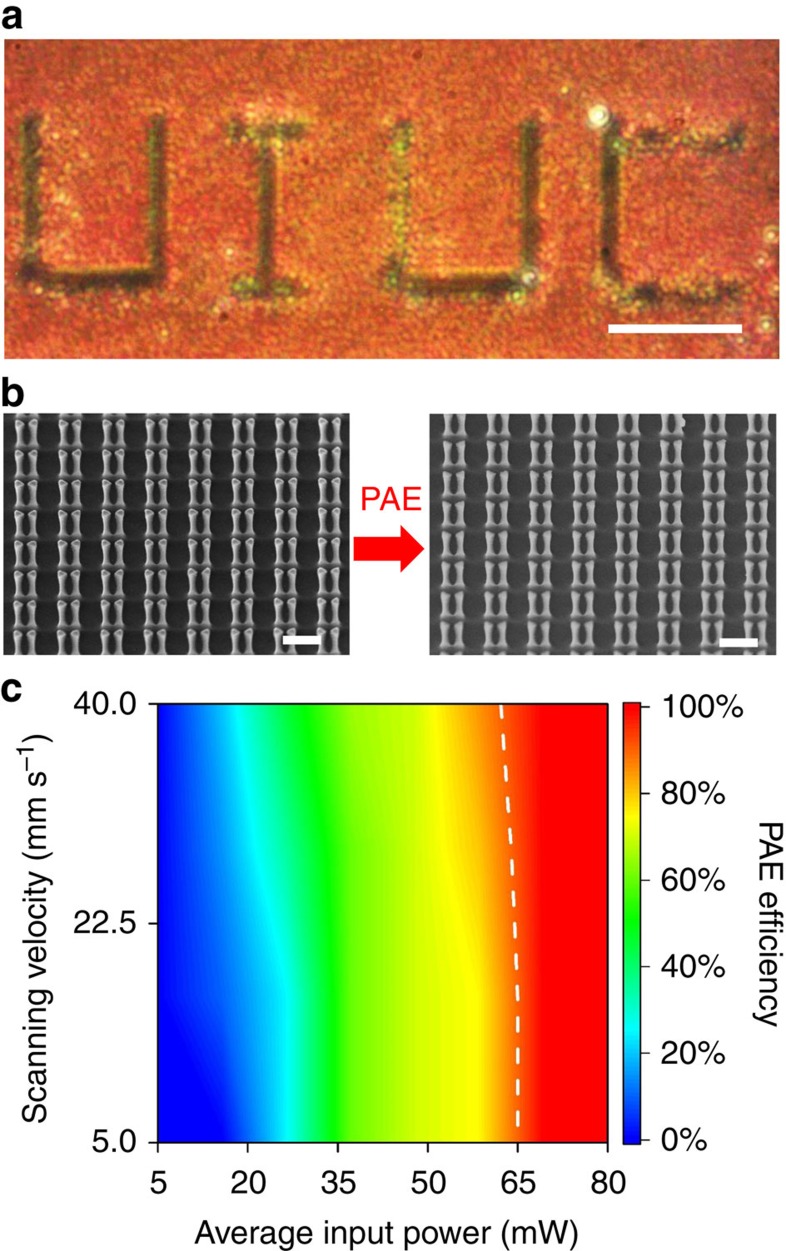
PAE process. (**a**) Dark-field image of the letters ‘UIUC' etched in the pBNA chip. Shaded and orange regions are the etched and unetched areas, respectively. Scale bar, 20 *μ*m. (**b**) Example SEM images of an unetched (left) and etched (right) region. Scale bar, 500 nm. (**c**) Plot of the PAE efficiency as a function of input power and laser beam scanning velocity. A dashed white line indicates a PAE efficiency threshold of 90%.

Figure 2 provides an opportunity to take a closer look at the PAE process. A microscope stage is scanned for fixed focused laser illumination of the pBNA template, whereby the Au nanoantennas debond from silica only for the illuminated regions. Note that the beam could have been similarly scanned, but stage scanning was chosen for convenience. We use this approach to etch the initials ‘UIUC', as observed in the dark-field image shown in Fig. 2a. Scanning electron micrographs (SEMs) of the etched structure are shown in Fig. 2b for both etched and unetched regions of the pBNA chip. From these images it is clear that this process cleanly debonds the metal, leaving the silica pillars unaffected. To determine the effect of input optical power and scan velocity of the focused laser beam on the PAE process, we independently control these parameters for a fixed pBNA array area (10 × 10 *μ*m^2^) and subsequently examine the percentage of metal completely removed in this region. Figure 2c summarizes the results, where the colour used corresponds to the percent efficiency of the process. The white dashed line delineates the threshold at which the PAE efficiency is >90%. We observe that the PAE process has a stronger dependence on average input power than scanning velocity. What is not revealed in the plot is that we find debonding of the metal for some of the pBNAs for average input powers as low as 10 mW. However, due to common inhomogeneities resulting from the electron-beam lithography process, for example, subtle variations in nanoantenna gap size and radius of curvature, an average input power of 65 mW is required to achieve at least 90% PAE efficiency for most of the scanning velocities used.

A straightforward argument can be used to understand the PAE process. To begin, the pBNA structure is immersed in water and illuminated by a focused pulsed laser beam spectrally centred at a wavelength *λ*=780 nm. The excitation source is a 100-*fs* pulsed, 80-MHz repetition rate, Ti:sapphire laser focused by a 0.6-numerical aperture (NA) microscope objective. Upon optical illumination the metallic nanoantenna structures begin to generate heat via optical absorption, and the corresponding heat power can be estimated through^29^


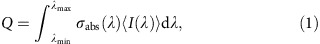


where *σ*_abs_(*λ*) is the spectral absorption cross-section of the metal layer of the illuminated pBNAs and *I*(*λ*) is the incident average intensity. The thermal conductivity ratio between the Au and the Ti (*k*_Au_/*k*_Ti_≈14) is much smaller than that between the Au and the surrounding water (*k*_Au_/*k*_water_≈512), and the Ti adhesion layer is firmly adhered to the gold bowties. In addition, the gold bowties have a significantly larger volume (∼10 × ) and exhibit larger optical absorption than their Ti adhesion layers. Thus, most of the heat generated is in the gold bowties, and the temperature increase is assumed to be uniform throughout the metal layer. For pulsed illumination, the temperature increase in the bowties can further be estimated through^29^


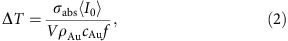


where *V* is the bowtie volume (0.0011 *μ*m^3^), *ρ*_Au_ is the density of gold (19,320 kg m^−3^), *c*_Au_ is the heat capacity of gold (129 J kg^−1 ^K^−1^) and *f* is the pulse repetition rate. This results in an absorption cross-section of 0.065 *μ*m^2^ for arrays of 525-nm spacing^30^. For input average powers near 90 mW, the metallic bowtie temperature can easily approach the melting point of bulk Au (∼1,064 °C), where surface melting near highly curved regions already happens^31^,^32^,^33^. When illuminated on resonance, the pBNA structure has stronger absorption and thus larger temperature increment than that illuminated off resonance as described by our simulation results in Supplementary Fig. 1. As a result of the heat generated from this optical absorption, both the metal nanoantennas and the SiO_2_ pillar at the interface undergo thermal expansion albeit with different thermal expansion coefficients. This effect leads to the generation of strain in the metal thin film, which later relaxes after the complete separation between the metal thin film and SiO_2_ pillar.

In addition to optical illumination, the ambient water alone plays an important role in the debonding process. Previous studies have shown that water can contribute to facile debonding of a metal film from a SiO_2_ substrate due to the strong polar interaction with the strained Si–O–Si crack-tip bonds^34^. In the context of the present work, Ti–O–Si bonds are believed to form during the e-beam deposition of the Ti adhesion layer. Once driven by external forces, the Ti–O–Si crack-tip bond reacts with water molecules to form Ti–O–H and Si–O–H bonds on each side of the separated interfaces and this process reduces the critical energy release rate required for delamination of the metal layer from the SiO_2_ pillar. This mechanism has been referred to as water-assisted subcritical debonding, and has been used in applications such as the peel-and-stick process^34^.

The delamination of the Au layer from the pBNA structure can be understood through an energy framework based on the Griffith criterion^35^. In this framework, strain energy reduction in the Au layer provides a configurational force that promotes delamination. It is possible to derive a precise debonding criterion for some simple geometries; here, however, we can only estimate the relative tendencies. We assume that there is no significant energy dissipation due to plasticity in the Au layer, and that the geometry can be considered as a planar thin film with a straight delamination front and no external loads. We then consider the question of whether relief of strain energy per unit area of delamination of the Au layer, or the energy release rate, is sufficient to overcome the work of adhesion in the film/substrate interface. The work of adhesion, Γ_0_, is given by^36^


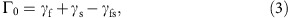


where *γ*_f_ and *γ*_s_ are the characteristic surface energy densities for the thin film and pillar substrate materials, respectively, and *γ*_fs_ is the interfacial characteristic free energy. Using values of the surface energy densities and interfacial free energies found in the literature^37^,^38^,^39^,^40^,^41^,^42^, we estimate Γ_0_ to be 1.8 J m^−2^ in the presence of water. This value suggests that spontaneous debonding of the thin film is highly unlikely without the heating due to laser illumination.

The work of adhesion is then compared with the strain energy density per unit area of the interface, or the energy release rate, which is given by^36^


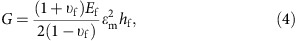


where the properties of the thin film are expressed in terms of Young's modulus *E*_f_, Poisson ratio 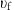, strain *ɛ*_m_ and thickness *h*_f_. The strain in the thin film is equal to (*α*_f_−*α*_s_)Δ*T*, where *α*_f_ and *α*_s_ are the linear thermal expansion coefficients for the thin film and pillar substrate respectively, and Δ*T* is the temperature increase. This expression assumes that the film is much thinner than the substrate, and that the delamination front is straight, which is clearly not perfectly valid here. Nevertheless, we arrive at an estimate of the energy release rate of ∼0.9 J m^−2^ for input average powers near 90 mW (refs 31, 32, 33).

We may then conclude that while spontaneous delamination in water is not likely, the application of the thermal strain due to the laser heating provides a significant driving force that is on the order of magnitude of the work of adhesion. Factors such as the three-dimensional nature of the strain, possible variations in the interface quality, and uneven heating, all may provide conditions that could lead to delamination under the laser heating conditions. Furthermore, the likely reduction of the strain energy density near the edges of the pillar, where the thermal mismatch strain is partially relaxed by the free surface, may be offset by the reduction of the critical strain energy release rate due to the chemical effects of the water environment, as noted above.

### Using PAE to fabricate basic planar, optical components

**Figure 3:**
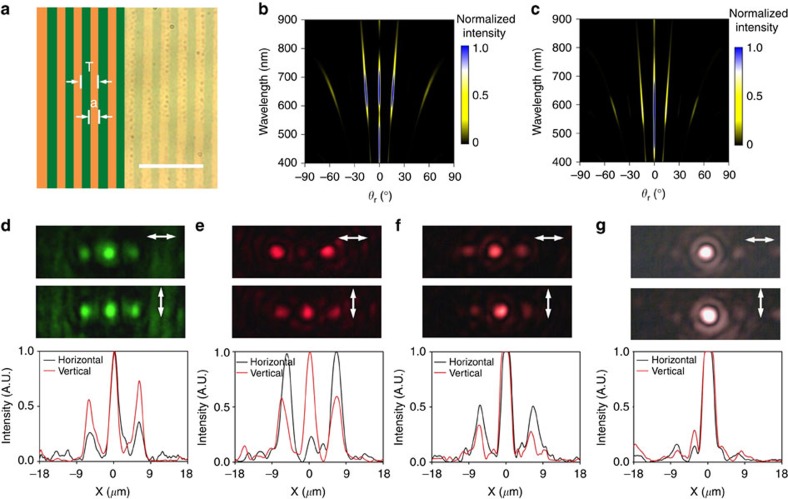
PAE-fabricated planar diffraction grating. (**a**) The schematic representation of the grating structure on the left with relevant parameters noted is overlaid with the bright-field image of a fabricated diffraction grating on the right. Scale bar, 20 *μ*m. In **b**,**c** we have the simulated normalized reflected intensity, for normally incident horizontal and vertical input polarization, respectively, as a function of input wavelength *λ* and the diffraction angle *θ*_r_. (**d**–**g**) Experimentally obtained intensity distributions and the corresponding cross-sectional intensity profiles for illumination wavelengths of 543, 660, 685 and 785 nm, respectively. For each case, the polarization state of the incident beam is indicated in the top right corner by the arrows.

As shown in Fig. 3a, PAE is used to fabricate a diffraction grating with a period *T* of 10 *μ*m and duty cycle of 50%. The yellow regions of the grating are the etched areas, displaying the colour of the glass substrate, while the unetched areas exhibit a green hue due to the gold antennas. To estimate the performance of the grating, we first employ FDTD simulations to numerically solve for the normalized reflected intensity as a function of input wavelength *λ* and diffraction angle *θ*_r_, when the grating is illuminated by normally incident light for either the *x*- (along the long bowtie axis) or *y*- (orthogonal to the long bowtie axis) polarization direction, as shown in Fig. 3b, c and Supplementary Figs 2 and 3, respectively. It is found that because of the plasmonic response of the structure, the grating effect emerges in the wavelength range of ∼600–800 nm for *x*-polarization and ∼500–620 nm for *y*-polarization, whereby ∼60% and 35% of the incident light are reflected at resonance, respectively. Within these bands of wavelengths, light is reflected back periodically at the surface of the pBNA chip making the component work as an amplitude grating. Outside the active wavelength regions, the diffraction grating behaves as a normal silica glass showing no diffraction. Thus, this type of structure can be used to route selected wavelengths, while leaving light at other wavelengths unaltered, particularly for applications related to ultra-compact optical systems where frequency demultiplexing is important. Further details on the characterization and simulation results are provided in Supplementary Figs 4–6. The experimentally measured diffraction patterns and associated cross-sectional intensity distributions are shown in Fig. 3d–g for laser wavelengths of 543, 660, 685 and 785 nm. At 660 nm, 65% of the light is concentrated into the first-diffracted order for the *x*-polarization, thereby behaving more like a blazed grating. In contrast, at 785 nm, most of the energy remains in the zeroth-order. Moreover, as shown in Supplementary Fig. 4, an increasing displacement of the first-order with respect to the wavelength is observed. The behaviour of our pBNA-based grating can be attributed to the wavelength selectivity of the plasmonic response. In this case, the dispersion relation is modified by the spectral envelope of the pBNAs. With incident wavelength approaching resonance, reflection of the unetched area is increased while the reflection from the etched area is kept the same. This effect leads to an increased diffraction efficiency towards resonance, as shown qualitatively in Fig. 3d–g and quantitatively in Supplementary Fig. 5. Note that the steering angle of the grating increases for longer wavelengths, as this quantity is determined by the diffraction equation *T*sin*θ=nλ*, where *T* is the grating period. In terms of the diffraction efficiency, we observe that for the vertical polarization the strongest is at 543 nm and it decreases as the wavelength increases, whereas for the horizontal polarization the strongest is ∼660 nm; the overall shape of the measured efficiency spectrum qualitatively agrees with the predicted spectrum, as shown in Supplementary Fig. 5. On the basis of our experiments, it is clear that although there is a phase contribution due to the plasmon resonance from the pBNAs, our fabricated components behave as ‘amplitude-mostly' elements.

**Figure 4:**
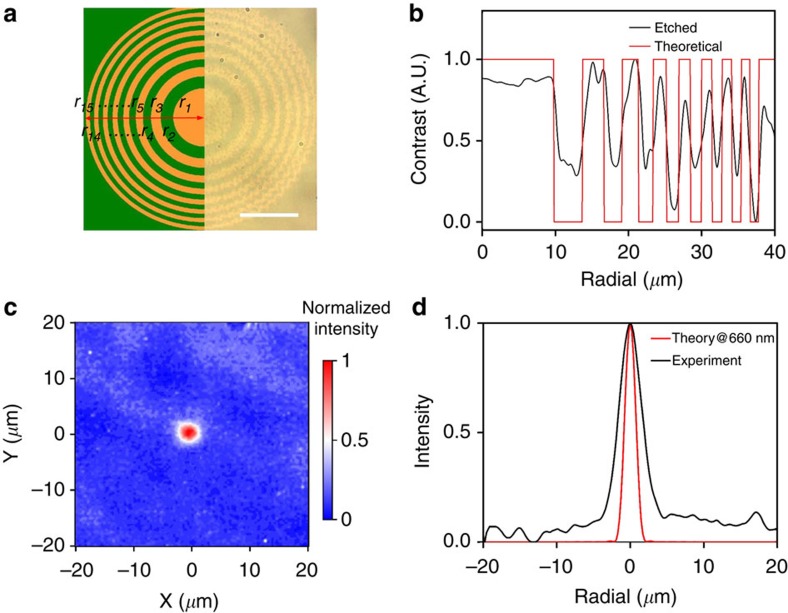
PAE-fabricated planar Fresnel zone plate. (**a**) Bright-field image of a fabricated Fresnel zone plate with overlaid schematic of the theoretical design. Scale bar, 10 *μ*m. (**b**) Comparison of theoretical (red) and experimental (black) contrast along the radial direction. (**c**) Experimentally obtained image of the focused intensity for illumination by a broadband source illumination. (**d**) Comparison of the theoretical (red) and experimental (black) focal-field intensity for laser illumination with wavelength of 660 nm.

In addition, it is also possible to use PAE to fabricate a FZP, as shown in Fig. 4a, with the bright-field image of the actual fabricated pattern overlaid with the schematic representation. The 80 × 80-*μ*m^2^ area of the pBNA chip is divided into 15 alternate concentric circles of etched and unetched regions. The width of each Fresnel zone is governed by the equation: 
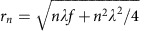
, where *n* is an integer, *λ* is the wavelength of the light which the FZP is designed for and *f* is the designed focal length of the FZP. We set the focal length to 150 *μ*m at an optical wavelength of 660 nm, the spacings and widths of the Fresnel zones are calculated with different values of *n* (*n*=1, 2, 3,…,15). In our particular case, we have 15 alternate zones on a chip. The stepwise edge of rings for the high order *n* is attributed to the coarse step size in the movement of galvo mirrors. Figure 4b shows the measured contrast for each zone in comparison with the theoretical value. The radius of the central zone is 10 *μ*m, and the lens radius is about 40 *μ*m. To demonstrate the lensing effect, we measure the cross-sectional intensity distribution in the focal plane with a plane wave broadband source illumination of the PAE-fabricated FZP, and the result is shown in Fig. 4c. A simulated intensity profile with a monochromatic visible light (660 nm) focused by a conventional lens (focal length of 150 *μ*m) is shown in Fig. 4d for comparison. Because of the fact that a broadband light source is used in the experiment whereas a monochromatic source is used in simulation, the lateral width of the experimentally measured focal spot is larger as it is a net combination of many focal points produced by different wavelengths. The detailed description of the optical system used for characterization is explained in Supplementary Fig. 7.

**Figure 5:**
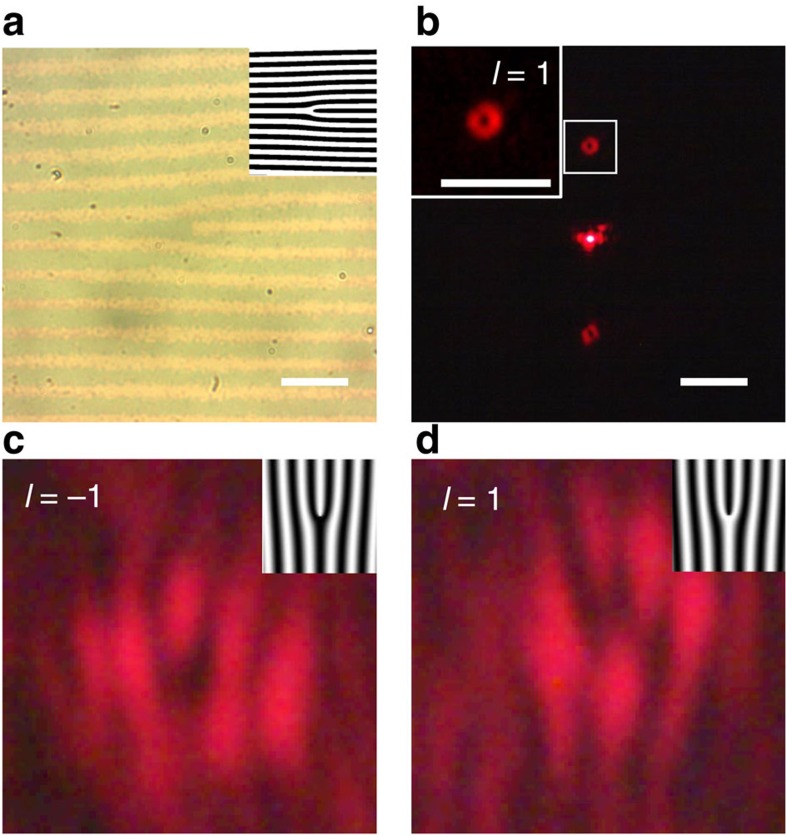
PAE-fabricated holographic pitch-fork pattern. (**a**) Bright-field image of a fabricated pattern. (**b**) Experimentally obtained optical vortices generated at the focal plane. Two donut beams of ±1 order are shown on either side of the central, zeroth-order beam. The inset shows a zoomed-in view of the +1 vortex. (**c**) Intensity distribution obtained from interfering a plane wave with the *l*=−1 beam, and (**d**) the *l*=+1 beam. The insets show the calculated fork grating design. Scale bar, 20 *μ*m.

We next use PAE to fabricate a fork dislocation grating via the optical set-up in Supplementary Fig. 8 to produce an optical vortex, as shown in Fig. 5a where the inset represents the schematic. Passage of a plane wave through this holographic structure results in a beam that carries orbital angular momentum (OAM)^41^,^42^,^43^. Optical vortices have been widely studied and play an important role in optical communications and particle trapping^44^,^45^,^46^,^47^. Generally, a spatial light modulator or a special organized liquid crystal display encoded with the computer-generated hologram of a ‘fork' is used to impart OAM. Our PAE-fabricated fork grating has a period of 10 *μ*m, a 50% duty cycle and a topological charge *l*=1. Weakly focused light is used to illuminate the fork dislocation grating, resulting at the focal plane with zero and ±1 diffraction orders as shown in Fig. 5b. As expected, the donut-shaped beam is generated in the ±1 diffraction orders due to a phase singularity at the centre. Note that both ±1 diffraction orders carry the same topological charge but opposite in sign. To extract the topological charge information for the diffracted order, the donut-shaped focal spot is interfered with a plane wave. The resulting patterns are shown in Fig. 5c,d for *l*=−1 and 1, respectively, where the forklet in the interference pattern indicates the helical phase embedded in the diffraction orders. The experimental results agree with the simulated interference pattern, and the estimated diffraction efficiency for the fork dislocation grating is ∼20%.

### Application to nanotweezers

In addition to fabricating flat optical components, PAE is also a useful approach to locally shape the trapping landscape of the nanoantenna array. Plasmonic optical trapping has become a popular application of nanoantennas. The enhanced electromagnetic-field confinement offered by nanoantennas enables efficient trapping of micro and nano-objects using low-input optical power densities^48^,^49^,^50^. We have shown previously that plasmon-induced heating effect can result in an alteration of the plasmon resonance of the pBNAs by photothermally changing the morphology of the Au nanoparticles. We showed that this effect could be used to tune the local potential energy landscape of the pBNAs^51^,^52^. In our current work, PAE provides a method to selectively etch out the gold nanoantennas and, thus, form inactive trapping regions. PAE results in zero net optical trapping force at the etched areas leaving unetched areas unaffected. Consequently the trapping effect is more robust in the PAE-fabricated channels since a deeper potential well is created compared with that done by plasmon-assisted heating^12^. Furthermore, optofluidic channels etched by PAE can be made in real time and subsequent optical trapping can be performed in the same aqueous solution^12^,^53^.

**Figure 6:**
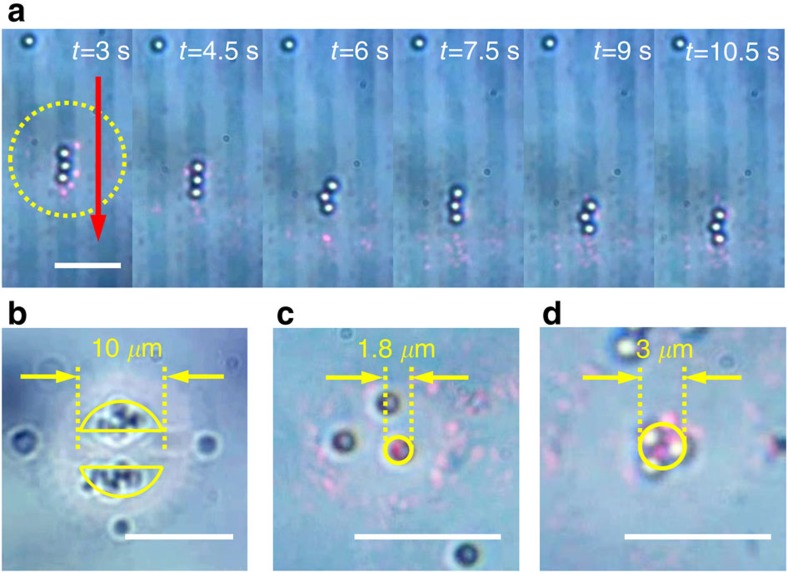
Using PAE to shape the trapping landscape. (**a**) Selected frames from a video demonstrating the guiding of 1-*μ*m diameter SiO_2_ particles in a predefined grating-like channel etched into the pBNA chip. The yellow circle and red arrow indicates, respectively, the optically illuminated region and direction of motion of the approximately collimated beam. Scale bar, 15 *μ*m. (**b**) Passive separation of microspheres into two crescent-shaped regions. (**c**) Demonstration of microspheres conforming to a predefined circular trapping region of 1.8-*μ*m diameter, and (**d**) 3-*μ*m diameter. Scale bars in **b**–**d**, 10 *μ*m.

To demonstrate the effect of our approach on plasmonic trapping, we apply PAE to create predefined trapping areas using ∼35.4 mW *μ*m^−2^ of intensity at the focal plane. As a result, gold nanoantennas with 35-nm gap size are removed from the silica pillars in the exposed area and preserved at the unexposed area. These unexposed gold nanoantennas provide a large trapping force at resonance of ∼0.02 pN. Specifically, we fabricate three kinds of predefined trapping patterns: a grating pattern of several line-shaped channels, a pattern of two adjacent crescent-shaped channels with an ∼5-*μ*m radius and a 2.5-*μ*m-wide isolation belt, and a pattern of a circular channel of two different radii. For trapping, a water-based colloidal suspension of 1-*μ*m-diameter SiO_2_ particles is injected into the water solution. Each fabricated pattern is illuminated with an approximately collimated, 25-*μ*m-diameter excitation beam obtained by focusing a 660-nm, horizontally polarized CW laser beam using a 0.6-NA objective. It is observed that particles are trapped merely in the predefined channels for all patterns as shown in Fig. 6. For the pattern of line-shaped channels, three particles in a chain are confined in a narrow channel as observed in Fig. 6a. Despite the activation of the next predefined channel which is 5 *μ*m away in distance, all particles remain in a chain only within the single channel, proving the existence of a sharp potential gradient at the edge of the channel. Next, by translating the sample stage vertically, and hence the pBNA chip, the particles move downward, in the opposite direction, as shown in Fig. 6a (Supplementary Movie 1). When the crescent-shaped channels are illuminated, a cluster of particles is dragged towards the trap by convection and redistributed in the shape of an isolated crescent (Fig. 6b and Supplementary Movie 2). Once all the particles become stabilized, the separation between two clusters is clearly observed. Moreover, as depicted in Fig. 6c (Supplementary Movie 3) and Fig. 6d (Supplementary Movie 4), the predefined trapping area can be reduced in size, so that fewer number of particles are allowed to be trapped until eventually single-particle trapping is achieved.

### A route to doubly heterogeneous nanoantenna arrays

**Figure 7:**
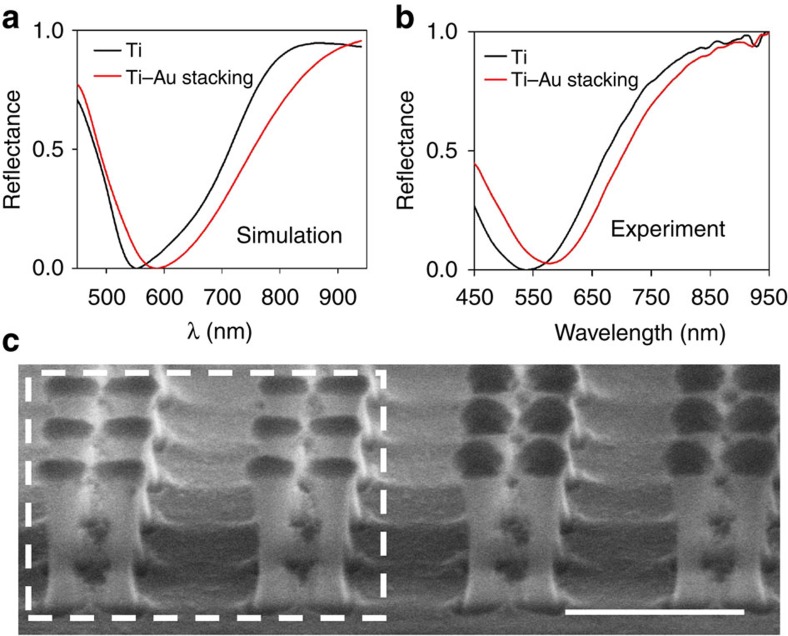
Metal stacking. (**a**) Simulated and (**b**) experimental reflection spectra for Ti pBNAs (black) and stacked Ti-Au pBNAs (red). (**c**) Corresponding SEM images of Ti pBNAs (in dashed white box) and Ti-Au pBNAs. Scale bar, 500 nm.

The results in Fig. 7 demonstrate another great advantage of the pBNA platform and PAE—the flexibility in creating doubly heterogeneous nanoantenna arrays. The illumination system discussed above is focused at the plane of nanoantenna arrays to scan the left half region of the 80 × 80-*μ*m^2^ pBNA chip. After applying PAE, we deposit a 50-nm layer of Ti onto the entire pBNA template through e-beam evaporation and thus successfully fabricate doubly heterogeneous nanoantenna arrays, where the left half region that is etched consists of nanoantenna arrays with a 50-nm Ti layer on SiO_2_ pillars, while the right half region that is unetched consists of nanoantenna arrays with a 50-nm Ti layer stacked on a 50-nm Au layer that sits on SiO_2_ pillars. We investigate the optical response of our etched and unetched regions, as shown in Fig. 7a,b for simulation and experiment, respectively. The optical response of unetched and etched areas are assessed by measuring the spectral reflectance *R*=1-*R*_raw_/max(*R*_raw_), where *R*_raw_ is the raw reflectance obtained by focusing a white-light source onto modified regions. We observe that the reflectance of the pBNAs with the single Ti layer exhibits a dip ∼550 nm, while that of the metal-stacking pBNAs exhibits a redshifted-dip ∼590 nm; note that both are blue-shifted compared with the original gold pBNAs before PAE. In the SEMs shown in Fig. 7c, the left two columns of the pBNA structure represent the scanned etched area, where the dark regions on top of the silica pillars indicate the 50-nm single layer of Ti. The right two columns of the pBNA structure represent the unetched area where the metal-stacked pBNAs are successfully fabricated with a 50-nm Ti layer deposited on top of a 50-nm Au layer. The physical appearance at the boundary between the etched and unetched areas of the pBNA arrays is clearly distinguishable under SEM. However, as seen in Fig. 7c, the shape of the second layer of Ti cannot precisely replicate that of the first layer of Au, as Ti accumulates on the side wall of the Au layer as well. The uneven height of the second layer of Ti and the change in the radius of curvature of the nanoantennas attribute to the slight discrepancy in the reflectance curves we observe between simulated and experimental results. Nonetheless, in this case, PAE provides an extra degree of freedom in manipulating the optical properties of such fabricated planar optical components.

## Discussion

As a step towards the realization of DIY optical components, we have demonstrated a novel approach to fabricating a class of planar optical components based on a template structure that consists of two-dimensional arrays of gold pBNAs. The uniqueness in our approach is the use of PAE offered by the nanoantennas such that table-top debonding of the gold from the silica pillars can be realized via laser-scanning optical microscopy. Therefore, by simplifying the steps from design-to-fabrication-to-application, PAE could introduce DIY planar optical components to a broad community of researchers who may not necessarily be specialist in nanophotonics, yet alone metasurfaces design. This would especially be true when large-scale, nanomanufacturing technologies^54^,^55^ are utilized in lieu of electron-beam lithography. As part of this study, we have shown the feasibility of utilizing the pBNA template and PAE to fabricate various kinds of optical components including a diffraction grating, FZP, and a holographic mode converter. In addition, we experimentally demonstrated how the pBNA template can be used to spatially tailor the optical potential energy landscape and thus enable preferential trapping and sorting of particles, which offers the possibility for fabricating optofluidic channels ‘without walls.' Moreover, the heterogeneity such as material composition and geometry within the pBNA template can also be controllably modified, thereby enabling a promising approach to readily tuning the optical response, such as the dispersive characteristics, of two-dimensional nanoantenna-based surfaces locally.

## Methods

### Fabrication

The surface bounded bowtie nanoantennas (BNAs) are patterned by electron-beam lithography on 5-nm ITO-coated glass substrates. For patterning the BNAs, a 100-nm-thick PMMA electron-beam resists layer is dispensed on the substrate and baked at 200 °C for 2 min. After exposure with 100 uC cm^−2^, the resist is developed in IPA:MIBK 3:1 for 45 s, rinsed with isopropyl alcohol for 30 s and dried under a stream of high-purity nitrogen. Using electron-beam evaporation technique, a 5-nm-thick Ti adhesion layer, a 50-nm-thick Au layer followed by an 8-nm Ni layer is deposited, respectively. After deposition, excess metal is removed by soaking the sample in acetone for 45 min. Finally, the pBNA structure fabrication is completed by performing reactive ion etching for 21 min with 70-s.c.c.m. CF_4_, 35-mtorr pressure and 90-W power. The fabricated pBNAs have 35-nm gaps with a 525-nm array spacing and pillars with a height of 500 nm. Note that on a single substrate we have 128 sample regions (pBNA chips). Fabrication of new optical elements is achieved simply by translating under the microscope to one of these new regions.

### Numerical simulations

Far-field reflectance spectra are calculated using the commercial FDTD software Lumerical FDTD-Solutions. The reflectance and transmittance of nanoantennas arrays are obtained by numerically solving Maxwell's equations under normal incidence plane wave source with polarizations parallel and perpendicular to the long antenna axis. In all simulations, the gold nanoantennas arrays supported by 500-nm SiO_2_ pillars are placed on a SiO_2_ substrate and situated at least one wavelength away from the edges of simulation box. Periodic boundary conditions and perfect matched layers are applied in the *x–y* plane and *z* direction (along the light propagation direction)^56^. The dielectric function of gold is taken from Johnson and Christy^57^. To resolve the nanostructure, the antennas and pillars are discretized into 3 × 3 × 3-nm^3^ meshes. Its reflectance and transmittance are calculated by integrating the power flux through a power monitor situated in air 1 μm above and below the sample plane, then normalizing it with respect to the source power.

### Microscope-based PAE

The beam used for PAE is derived from a Ti:sapphire laser (Spectra Physics Mai Tai) and polarized along the horizontal axis of the pBNA structure. Before coupling to the microscope, the laser beam is reflected by a pair of galvo mirrors which are operated for beam steering by Labview (National Instruments Corporation), a data acquisition and instrument control platform^58^. The galvo driver is connected to a DAQ board (NI USB-6221) with the position of the mirrors controlled by the output voltage. A 0.6-NA, collar-adjustable microscope objective (Olympus LUCPlanFLN × 40) is used to focus the incident laser beam onto the plane of the pBNA structure, which is placed on the sample stage of a standard microscope. A white-light source (Ocean Optics HL-2000) with an approximate bandwidth over 400–1,000 nm is used to measure the reflectance of the doubly heterogeneous nanoantenna arrays.

## Additional information

**How to cite this article:** Chen, H. *et al.* Towards do-it-yourself planar optical components using plasmon-assisted etching. *Nat. Commun.* 7:10468 doi: 10.1038/ncomms10468 (2016).

## Supplementary Material

Supplementary Information Supplementary Figures 1-8.

Supplementary Movie 1 Three particles are preferentially trapped and move downward within the predefined channel via PAE.

Supplementary Movie 2 Particles are preferentially trapped within two predefined crescent-shaped channels via PAE. The separation between two isolated crescent regions is clearly observed between the two clusters of trapped particles.

Supplementary Movie 3 Single-particle trapping in a predefined area of reduced size.

Supplementary Movie 4 Fewer particles are preferentially trapped within the predefined channel smaller than that in Supplementary Movie 2.
