# Supplementary material for: Towards do-it-yourself planar optical components using plasmon-assisted etching
Source: Nat Commun. 2016 Jan 27;7:10468. doi: 10.1038/ncomms10468 (PMC4737853; doi:10.1038/ncomms10468)
Supplement: Supplementary Information — Supplementary Figures 1-8. [file ncomms10468-s1.pdf]

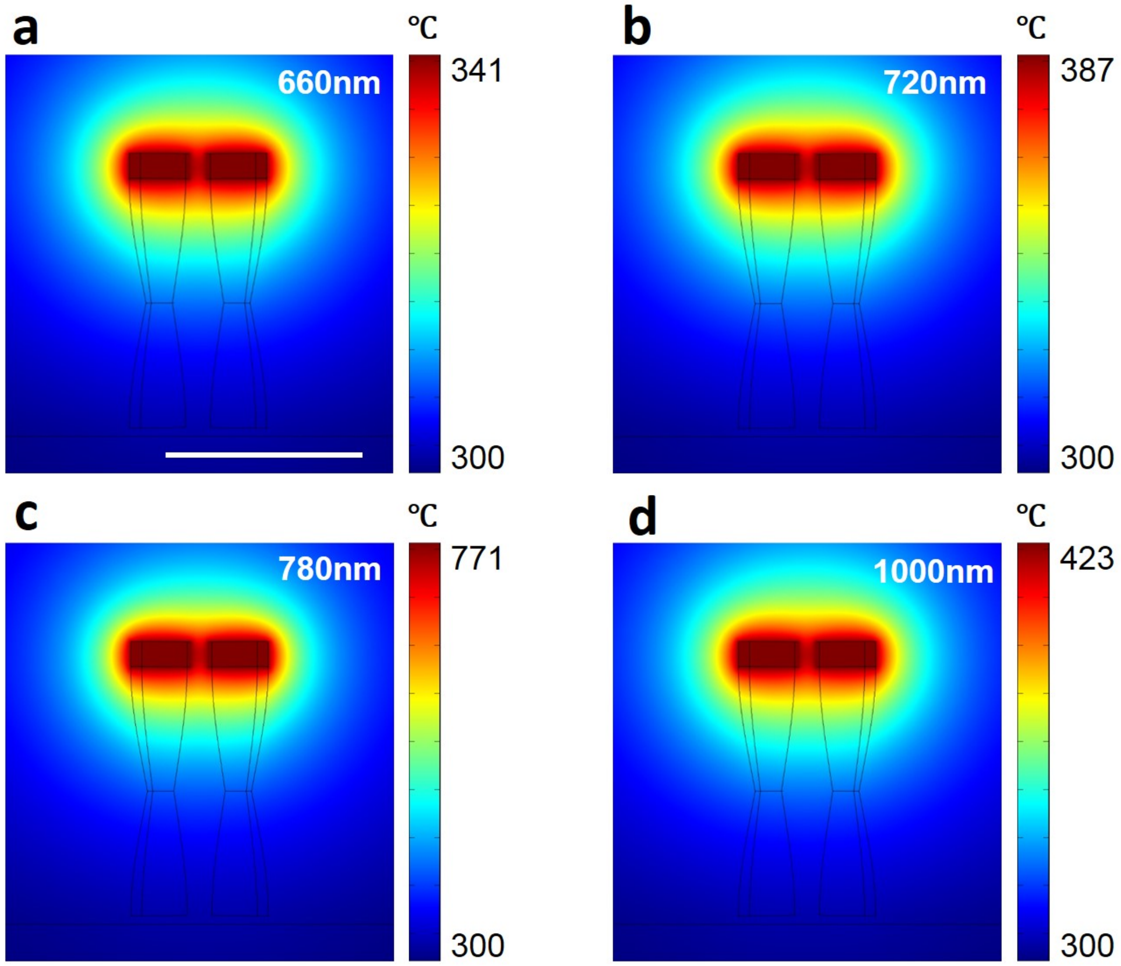

Supplementary Figure 1| 2D plot of simulated temperature distribution of pBNAs. Optical power is used to illuminate pBNAs is 10mW at wavelength of (a) 660 nm, (b) 720 nm, (c) 780 nm and (d) 1000 nm. The white scale bar represents 400 nm.

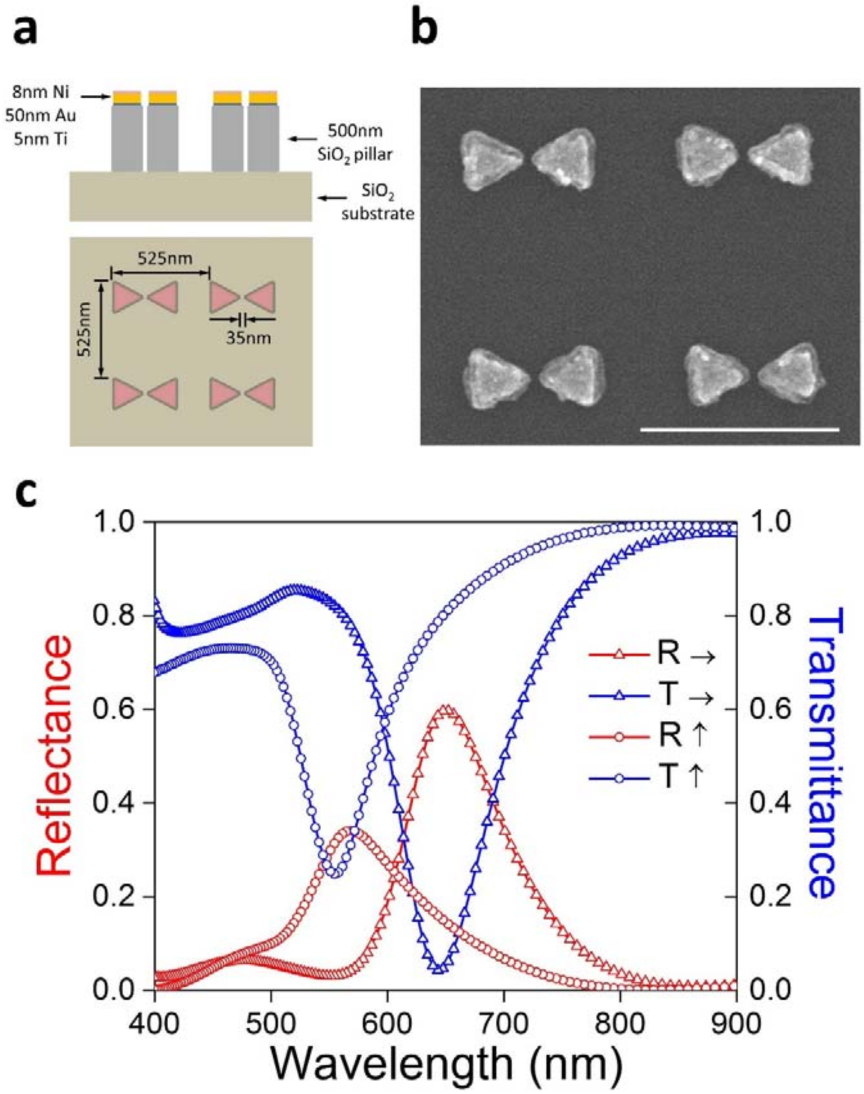

Supplementary Figure 2| Property of pBNAs. (a) Schematics of pBNAs (top view and side view) showing the dimensions of an individual pillar-supported bowtie nanoantennas and array spacings. (b) SEM images of pBNAs template (top view). The scale bars represent 500 nm. (c) FDTD Simulated normalized reflectance (red) and transmittance (blue) of the pBNAs with horizontal (open triangle) and vertical (open circle) polarizations.

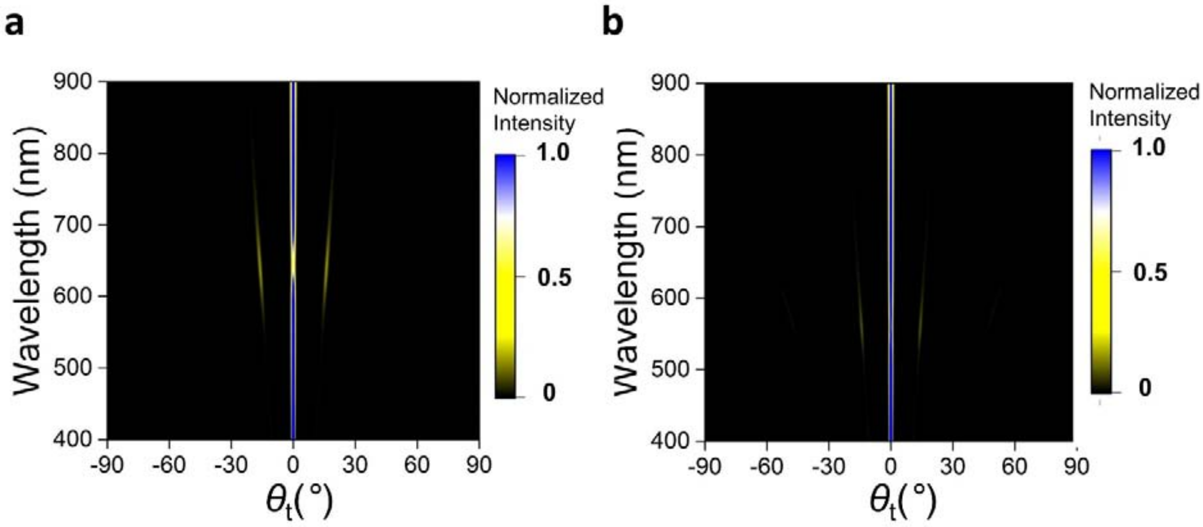

Supplementary Figure 3| Plots for transmission. FDTD simulated normalized transmitted field as a function of input light wavelength λ and the diffraction angle θ_t with (a) horizontal and (b) vertical polarizations.

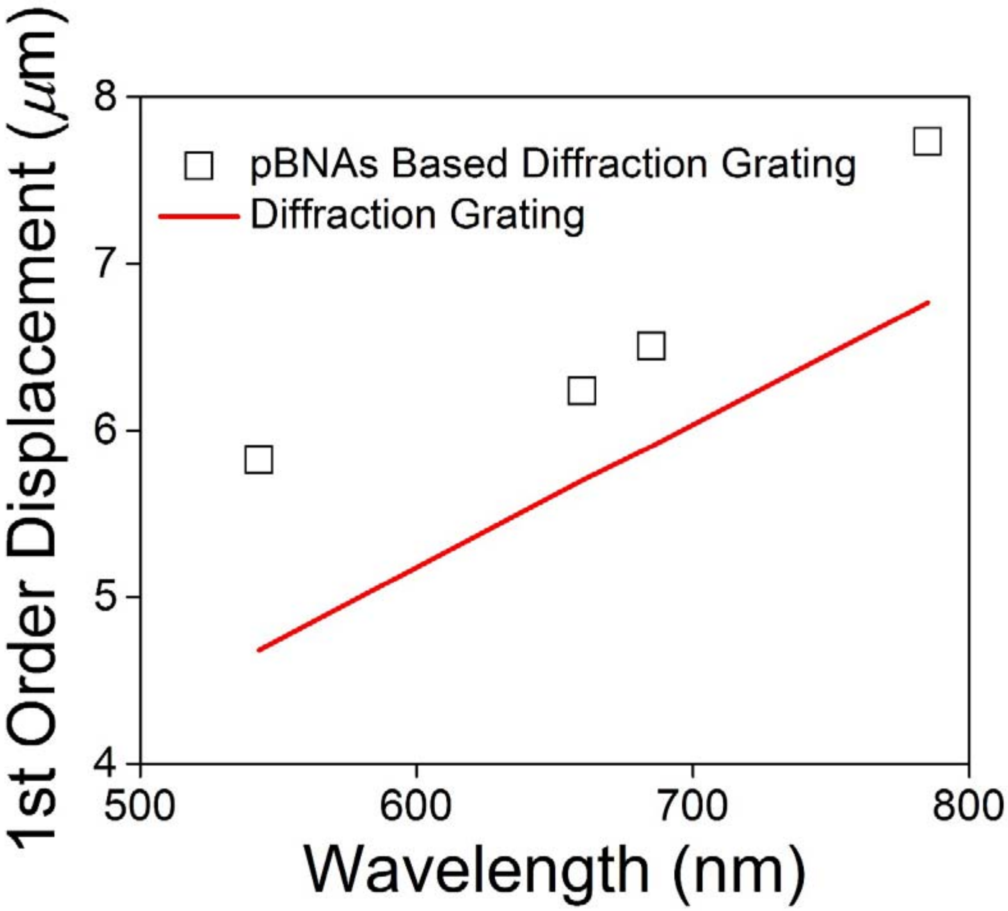

Supplementary Figure 4| Comparison of the measured (PAE based diffraction grating) (black square) and simulated (diffraction grating) (red line) displacement of the 1st orders for horizontal polarization scale as the wavelength.

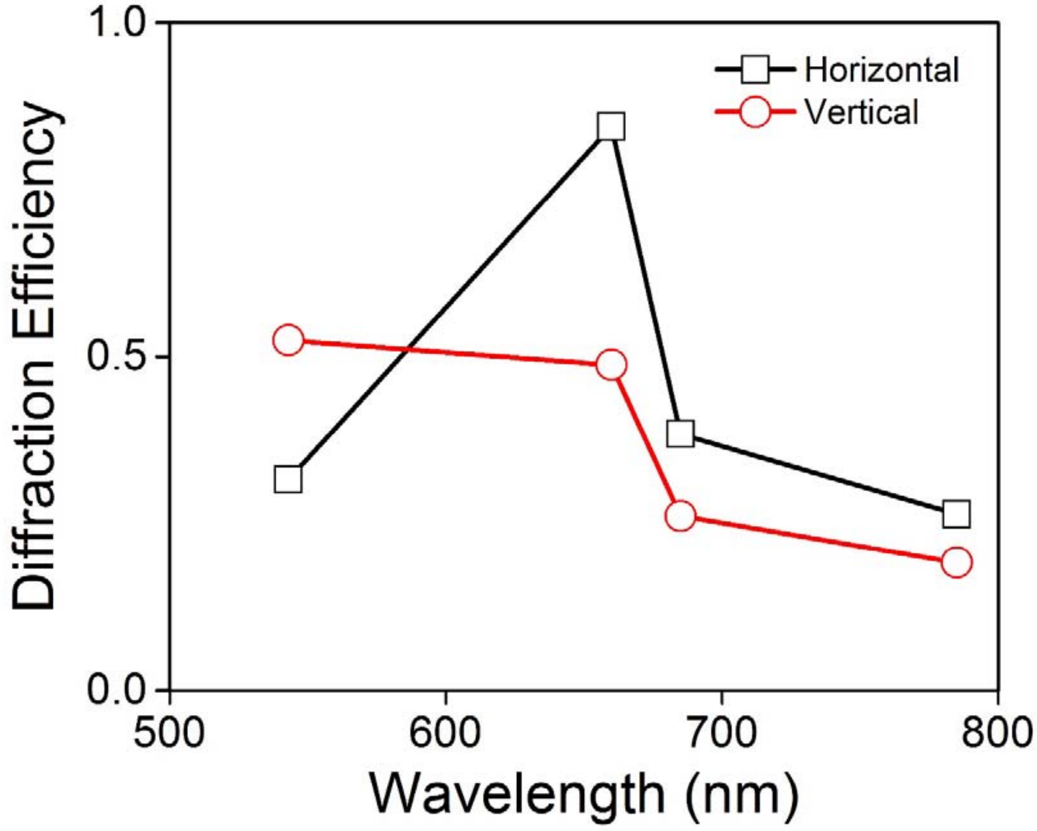

Supplementary Figure 5| Experimental diffraction efficiency as a function of input light wavelength λ with horizontal (black) and vertical (red) polarizations.

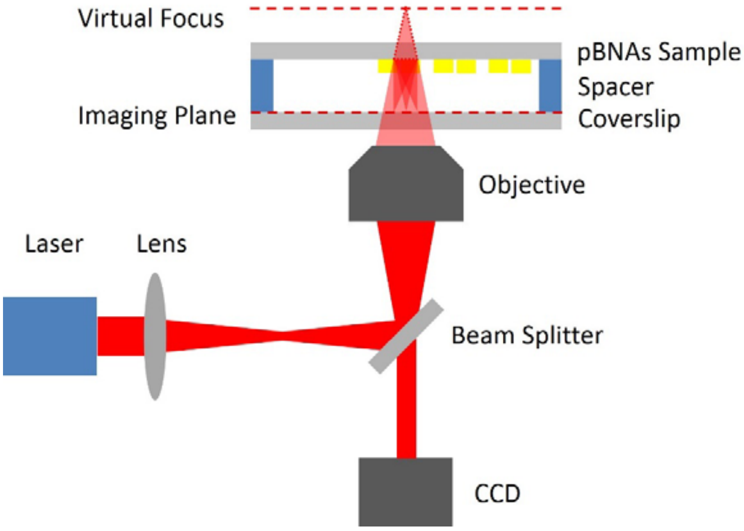

Supplementary Figure 6| Schematic of the experimental setup for measuring the far-field diffraction patterns from PAE-fabricated planar diffraction grating. The laser beam derives from a 660-nm wavelength continuous-wave laser diode. The optical system is constructed around a customized microscope. An objective (Olympus LUCPlanFLN 40x) is used to capture the image of the light reflected by the diffractive grating. The imaging plane of the CCD is adjusted to the focal plane of the objective. In such case, an additional tube lens outside of the microscope is used to tune the output beam wavefront of the objective, whereby the focal plane of the reflected light is adapted to the imaging plane. To do so, the diffraction pattern is captured by the CMOS color camera (Thorlabs, DCC1645C).

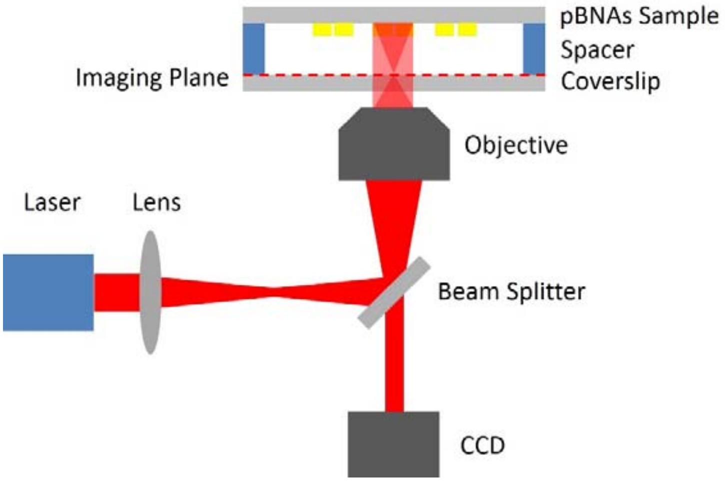

Supplementary Figure 7| Schematic of experimental setup for measuring the focus distribution of FZP fabricated by PAE. The illumination beam is obtained from a photonic crystal fiber (Femtowhite 800, NKT Photonics) pumped using a Ti:sapphire laser with 100-*fs* duration pulse width, 80-MHz pulse repetition rate, 800-nm center wavelength, and ~200-mW average power. A combination of tube lens and objective (Olympus LUCPlanFLN 40x) forming a 4f system is used to generate a collimated output beam to illuminate the FZP. A 0.15-mm thick coverslip is chosen as a spacer. The thickness is equivalent to the designed focal length of the FZP. The imaging plane of the microscope system is placed at the top surface of the coverslip on the bottom. Thereby, the CMOS camera is able to capture the image of the scattered field on the coverslip surface.

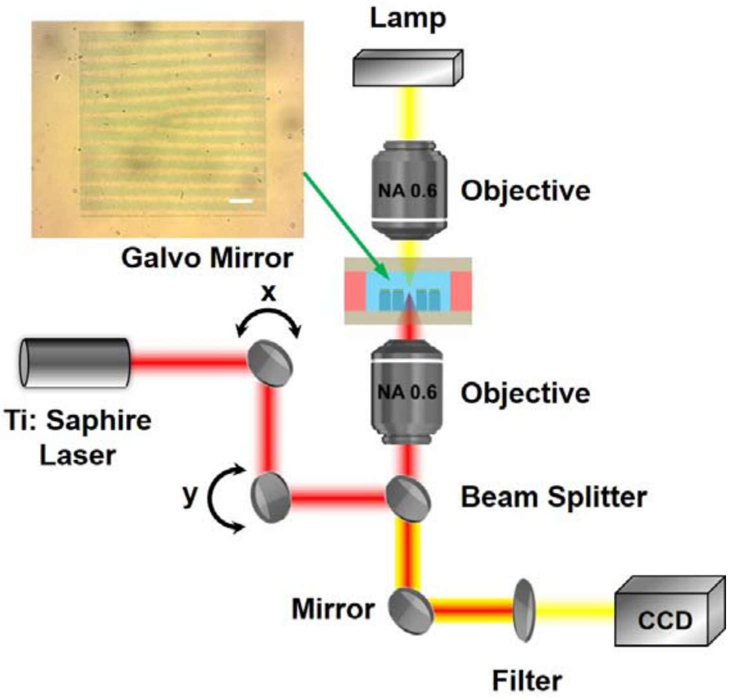

Supplementary Figure 8| Schematic of experimental setup and an inset image of the pBNAs chip including substrate after PAE.
